# Quercetin: a promising virulence inhibitor of *Pseudomonas aeruginosa* LasB *in vitro*

**DOI:** 10.1007/s00253-023-12890-w

**Published:** 2024-01-05

**Authors:** Yanying Ren, Rui Zhu, Xiaojuan You, Dengzhou Li, Mengyu Guo, Bing Fei, Ying Liu, Ximing Yang, Xinwei Liu, Yongwei Li

**Affiliations:** 1 Henan University of Chinese Medicine, Zhengzhou, 450046 China; 2 Henan Province Hospital of Traditional Chinese Medicine, The Second Affiliated Hospital of Henan University of Chinese Medicine, Zhengzhou, 450002 China; 3 The Key Laboratory of Pathogenic Microbes & Antimicrobial Resistance Surveillance of Zhengzhou, Zhengzhou, 450002 China; 4 Henan Engineering Research Center for Identification of Pathogenic Microbes, Zhengzhou, 450002 China; 5 Henan Provincial Key Laboratory of Antibiotics-Resistant Bacterial Infection Prevention & Therapy with Traditional Chinese Medicine, Zhengzhou, 450002 China; 6 Dongzhimen Hospital of Beijing University of Chinese Medicine, Peking, 100700 China

**Keywords:** Quercetin, *Pseudomonas aeruginosa*, LasB elastase, Quorum sensing, Antivirulence therapy

## Abstract

**Abstract:**

With the inappropriate use of antibiotics, antibiotic resistance has emerged as a major dilemma for patients infected with *Pseudomonas aeruginosa*. Elastase B (LasB), a crucial extracellular virulence factor secreted by *P. aeruginosa*, has been identified as a key target for antivirulence therapy. Quercetin, a natural flavonoid, exhibits promising potential as an antivirulence agent. We aim to evaluate the impact of quercetin on *P. aeruginosa* LasB and elucidate the underlying mechanism. Molecular docking and molecular dynamics simulation revealed a rather favorable intermolecular interaction between quercetin and LasB. At the sub-MICs of ≤256 μg/ml, quercetin was found to effectively inhibit the production and activity of LasB elastase, as well as downregulate the transcription level of the *lasB* gene in both PAO1 and clinical strains of *P. aeruginosa*. Through correlation analysis, significant positive correlations were shown between the virulence gene *lasB* and the QS system regulatory genes *lasI*, *lasR*, *rhlI*, and *rhlR* in clinical strains of *P. aeruginosa*. Then, we found the *lasB* gene expression and LasB activity were significantly deficient in PAO1 *ΔlasI* and *ΔlasIΔrhlI* mutants. In addition, quercetin significantly downregulated the expression levels of regulated genes *lasI*, *lasR*, *rhlI, rhlR*, *pqsA*, and *pqsR* as well as effectively attenuated the synthesis of signaling molecules 3-oxo-C12-HSL and C4-HSL in the QS system of PAO1. Quercetin was also able to compete with the natural ligands OdDHL, BHL, and PQS for binding to the receptor proteins LasR, RhlR, and PqsR, respectively, resulting in the formation of more stabilized complexes. Taken together, quercetin exhibits enormous potential in combating LasB production and activity by disrupting the QS system of *P. aeruginosa in vitro*, thereby offering an alternative approach for the antivirulence therapy of *P. aeruginosa* infections.

**Key points:**

• *Quercetin diminished the content and activity of LasB elastase of P. aeruginosa.*

• *Quercetin inhibited the QS system activity of P. aeruginosa.*

• *Quercetin acted on LasB based on the QS system.*

**Supplementary Information:**

The online version contains supplementary material available at 10.1007/s00253-023-12890-w.

## Introduction

*Pseudomonas aeruginosa* (*P. aeruginosa*), a Gram-negative bacillus widely distributed in various environments, is one of the most important opportunistic pathogens responsible for nosocomial and community-acquired infection (Jurado-Martín et al. 2021; Qin et al. 2022). Given the increasing morbidity and mortality worldwide, the classification of *P. aeruginosa* as an ESKAPE pathogen by the World Health Organization highlights the urgent need for novel antibiotics (De Oliveira et al. 2020). Currently, the clinical isolates of *P. aeruginosa* are mainly composed of multidrug-resistant (MDR) and extensively drug-resistant (XDR) strains, which pose serious challenges to clinical infection management and treatment (Horcajada et al. 2019; Pérez et al. 2019). Under such circumstances, there is an urgent need to develop new drugs and methods for the treatment of *P. aeruginosa* infections. In contrast to traditional antibiotic treatment which directly affects bacterial survival, an antivirulence strategy that targets bacterial virulence factors of pathogens to suppress bacterial pathogenicity has emerged as a new alternative in the treatment of *P. aeruginosa* infections (Galdino et al. 2019b; Pang et al. 2019).

Virulence factors from *P. aeruginosa* can facilitate bacterial infection by inducing pathological damage to the host and evading host immune attack, which plays an important role in the pathogenesis of *P. aeruginosa* (Jurado-Martín et al. 2021). As one of the most abundant extracellular virulence proteins secreted by *P. aeruginosa*, LasB elastase exhibits multiple biological functions, such as degrading host tissue proteins, inducing inflammatory responses, and impairing the host innate immunity, which contribute to the establishment and maintenance of *P. aeruginosa* infections (Everett and Davies 2021; Galdino et al. 2019a). Furthermore, LasB can also evade host immune defense and prevent bacterial clearance by degrading circulating immune molecules (e.g., immunoglobulins, cytokines, and complements), which can lead to chronic recurrent infections and even host death (Bastaert et al. 2018; Everett et al. 2023; Galdino et al. 2019a; Sun et al. 2020; Zupetic et al. 2021). Therefore, direct or indirect suppression of LasB may prevent LasB from invading host tissues or manipulate the immune defense system to some extent, alleviate the toxic effects of *P. aeruginosa*, inhibit infection establishment, and avoid persistent colonization of *P. aeruginosa* (Wagner et al. 2016).

A variety of mechanisms are involved in the regulation of bacterial virulence, of which quorum sensing (QS) is crucial (Letizia et al. 2022). The QS system is a complex means of intercellular communication in bacterial communities that exhibit density-dependent responses (Chadha et al. 2021). *P. aeruginosa* has three separate and closely interrelated subsystems, including the Las, Rhl, and *Pseudomonas* quinolone signal (PQS) systems (Chadha et al. 2021; Cornelis 2020; Kostylev et al. 2019). Las and Rhl systems are induced and activated by acyl-homoserine lactones (AHLs). Specifically, LasI signal synthase is responsible for synthesizing the autoinducer N-(3-oxy-dodecanoyl)-L-homoserine lactone (3-oxo-C12-HSL, OdDHL), which is involved in binding to the transcriptional regulator LasR. On the other hand, RhlI signal synthase is in charge of the synthesis of autoinducer N-butyl-L-homoserine lactone (C4-HSL, BHL) and subsequently interacts with the transcriptional regulator RhlR (Chadha et al. 2021; Kumar et al. 2015). As for Pqs, it is activated by the compound formed by the combination of the autoinducer 2-heptyl-3-hydroxy-4-quinolone (PQS) with the transcriptional regulator PqsR (García-Reyes et al. 2020). Finally, the complexes of autoinducer signal molecules and transcriptional regulatory proteins are involved in the transcription of virulence genes (including *lasB*) (Lee and Zhang 2015). The regulation process of the QS system is fundamental to understanding the molecular mechanisms underlying the *P. aeruginosa* pathogenicity. Interfering with this process is essential for the investigation and management of bacterial infections. Consequently, deleting or mutating genes in the QS system, inactivating or suppressing the activity of signaling molecules, and competitively binding with analogues of signaling molecules to transcriptional regulatory proteins can all help reduce the production and activity of LasB (Defoirdt 2018).

Monomers and low-molecular-weight compounds derived from Chinese herbs and plants are regarded as valuable resources for the development of novel therapeutics targeting *P. aeruginosa* infection (Almalki et al. 2022; Chadha et al. 2022a; Rather et al. 2021). Quercetin, a plant-derived flavonoid compound abundant in vegetables, fruits, and Chinese herbs, exhibits powerful antibacterial, anti-inflammatory, antioxidant, and antitumor effects (Yang et al. 2020). It has been reported that quercetin has a direct bacteriostatic effect by destroying bacterial structures, altering cell permeability, and inhibiting the binding of nucleotides to proteins (Nguyen and Bhattacharya 2022; Yang et al. 2020). Quercetin has also been shown to inhibit the production of bacterial virulence factors and the formation of biofilms (Ouyang et al. 2020; Ouyang et al. 2016). Due to the outstanding advantages of quercetin and the seriousness of *P. aeruginosa* infection, we first determined whether quercetin could inhibit the production and activity of LasB from *P. aeruginosa* and then evaluated the corresponding mechanism from the effects of quercetin on the QS system, hoping to illustrate the feasibility of quercetin as an antivirulence agent against LasB of *P. aeruginosa*.

## Materials and methods

### Bacterial strains

The clinical isolates of *P. aeruginosa* were collected from the clinical microbiology laboratory of Henan Provincial Hospital of Traditional Chinese Medicine from January 2022 to September 2023. The *P. aeruginosa* PAO1 (ATCC 15692/DSM 22644, Catalogue Number: Bio-82076) was a standard strain purchased from Beijing Biobw Biotechnology Co., Ltd. (Peking, China). All the strains were maintained in Luria-Bertani (LB) medium containing 25% (v/v) glycerol at −80 °C. After recovery on Columbia agar (Autobio, Zhengzhou, China), a single colony was inoculated into LB broth (Solarbio, Peking, China) for sub-cultivation at 200 rpm, 37 °C until OD600 nm reached 0.5 (1×10^8^ CFU/ml, approx.). The cultures were then diluted 1:100 (1×10^6^ CFU/ml, approx.) in LB broth for subsequent experiments.

### Materials

Quercetin, a reference standard with a purity of ≥ 98% (HPLC), was purchased from Solarbio Science & Technology Co., Ltd. (Peking, China). Given the noteworthy effectiveness of antibiotics in combating microorganisms and the established role of baicalin as a natural QS inhibitor (Luo et al. 2017; Zhang et al. 2021), we chose a baicalin reference standard with a purity of ≥ 98% (HPLC) from Yuanye Biotechnology Co., Ltd. (Shanghai, China) as a positive control. The 20 mg/ml of stocks of quercetin and baicalin was prepared in dimethyl sulfoxide (DMSO) at a final concentration of 2% (v/v) and stored at −20 °C until further use.

### Construction of ΔlasI and ΔlasI ΔrhlI-deficient mutants of PAO1

Homologous recombination was applied to construct *lasI* and *rhlI* gene defective strains of PAO1, which are responsible for the synthesis of Las and Rhl autoinducer synthases in the QS system. The required primers were synthesized by Sangon Bioengineering Co., Ltd. (Shanghai, China) and listed in Table S1. The gentamicin and ampicillin resistance gene fragments were amplified from the pUC57 plasmid. The upstream and downstream homologous arms of *lasI* and *rhlI* genes to be knocked out in PAO1 were fused to the gentamicin and ampicillin resistance gene fragments, respectively. Then, the fused fragment was cloned into the suicide plasmid pCVD442 to obtain the targeting plasmid. The targeting vector was electro-transformed into competent cells of *E. coli* DH5α λpir, and the positive clones obtained were screened by corresponding resistant agar plates and verified by sequencing. The validated recombinant vector was electro-transformed into *E. coli* β2155 to obtain the donor bacteria for binding experiments with PAO1 receptor bacteria and then the positive clones were screened on resistance plates. Clones *ΔlasI* (single gene deletion mutant) and *ΔlasIΔrhlI* (double gene deletion mutant) in PAO1 were obtained by 10% sucrose reverse screening and PCR.

### MIC determination

Based on Clinical and Laboratory Standards Institute (CLSI) guideline M07-A10 (CLSI 2018), the broth microdilution method was used to determine the Minimum Inhibitory Concentrations (MICs) of quercetin and baicalin against PAO1 (Xiao et al. 2012). The MIC was defined as the minimum effective concentration that inhibited bacterial growth and did not cause broth to turn significantly red, as indicated by 0.02% resazurin dye (Chadha et al. 2023).

### Time-kill assay

Growth curves were plotted according to Bose’s method with modifications (Bose et al. 2020). Briefly, PAO1 bacterial suspensions pretreated with quercetin or baicalin at different concentrations were cultured at 37 °C, 200 rpm, for 24 h. The bacterial suspension was collected at 2 h intervals from the onset of the culture and subjected to plate colony counting to determine the colony forming units (CFU) at the appropriate time. After 24 h of incubation, the bacterial density was detected spectrophotometrically at 600 nm.

### Determination of activity and content of LasB

Since LasB elastase in the culture supernatant of *P. aeruginosa* could hydrolyze the substrate elastin, a qualitative test of the enzyme activity of LasB was performed on the elastin agar plate (Alpha Chemical Co., Ltd., Zhengzhou, China, 9007-58-3). The visible size of the hydrolysis area somewhat represented the hydrolysis activity of LasB elastase.

Elastin-Congo red (ECR) (Sigma-Aldrich, SHBL9368) was used to determine the LasB elastase activity as previously published (Yang et al. 2021). Briefly, 500 μl of filtered supernatant was added to 500 μl of ECR buffer (100 mM Tris-HCl, pH 7.5) containing 20 mg ECR and shaken at 37 °C for 18 h. The reaction was terminated by adding 0.1 ml of 0.12 M EDTA. Insoluble ECR was removed by centrifugation at 12,000 rpm for 10 min, and the absorbance was measured at 495 nm.

The LasB activity was also determined using the EnzChek Elastase Assay Kit (Invitrogen, Carlsbad, USA, 2407850), following the method described by Santajit et al. (Santajit et al. 2021). The 96-well microplate was incubated in darkness at 37 °C, and the fluorescence intensity was monitored dynamically every 10 min for 2.5 h (Ex/Em = 485 nm/530 nm) using the Cytation™ 3 Cell Imaging Multi-Mode Reader (BioTek Instruments Co., Ltd., Vermont, USA). Background values were subtracted from the fluorescence intensity value of each well.

The Elastase ELISA KIT (Enzyme-Linked Biotechnology Co., Ltd., Shanghai, China) was used to determine the LasB elastase levels. The corresponding OD450 nm was determined using a PHOMO microplate reader (Autobio Co., Ltd., Zhengzhou, China).

### Conventional PCR

The amplification system for the *lasI*, *lasR*, *rhlI*, *rhlR*, and *lasB* genes was constructed using the SLAN-96P real-time fluorescence PCR instrument (Hongshi Medical Technology Co., Ltd., Shanghai, China), the 2⨯Taq PCR Master Mix Kit (Sangon Bioengineering Co., Ltd., Shanghai, China, #B929295-0100), and oligonucleotide primers listed in Table S2. The amplified products were detected by agarose gel electrophoresis and captured via the Tanon gel imaging system (Tanon Life Science Co. Ltd., Shanghai, China).

### RT-qPCR gene expression analysis

TRIzol™ Reagent (Sangon Bioengineering Co., Ltd., Shanghai, China, B511311-0100) was used to extract the total RNA from PAO1 cultures treated with varying concentrations of quercetin. Subsequently, MightyScript First Strand cDNA Synthesis Master Mix (Sangon Bioengineering Co., Ltd., Shanghai, China, G709KA6194) was used to perform reverse transcription of RNA into first-strand cDNA. Afterward, SYBR Green Fast qPCR Master Mix (Sangon Bioengineering Co., Ltd., Shanghai, China, B639271-0005) was employed for RT-qPCR amplification. Relevant specific primers are listed in Table S3. *16S rRNA* was chosen as the reference gene. By normalizing the cycle threshold (CT) of the target and reference genes, the 2^-△△Ct^ method was used to determine the expression levels of the target gene.

### SDS-PAGE electrophoresis

The bacterial culture was centrifugated to separate the supernatant and precipitate. The supernatant was filtered through a 0.22-μm sterile filter, while the precipitate was sonicated to release bacterial proteins. After SDS-PAGE, the proteins were stained with Coomassie brilliant blue and destained with a destainer (40% absolute ethanol + 10% glacial acetic acid). Visualization of both extracellular and bacterial proteins was performed using the Tanon gel imaging system (Shanghai, China).

### AHL determination

An ultrahigh-performance liquid chromatography-triple quadrupole-composite linear ion trap mass spectrometer (AB SCIEX 4000 Qtrap® LC-MS/MS, Massachusetts, USA) was used to determine the levels of OdDHL and BHL. Standards of OdDHL (Macklin Chemical Reagent, Shanghai, China, C14148475) and BHL (Macklin Chemical Reagent, Shanghai, China, C14056603) were used to construct standard curves and establish linear regression equations. The AHLs were extracted in isovolumetric ethyl acetate and dissolved in methanol. The actual concentration of AHLs in the sample was calculated by determining the dilution ratio and comparing the peak area of the standard with that of the corresponding retention time.

### Molecular docking

Computational molecular docking is extensively utilized for analyzing ligand-receptor binding interactions (King et al. 2016). AutoDock Vina 1.1.2 was used to verify the binding potential between compounds and key targets. The 3D structures of the natural signaling molecules (including OdDHL, BHL, and PQS) and the small molecule compound quercetin were downloaded from the PubChem database. The 3D structures of the receptor proteins LasB (PDB ID:7OC7), LasR (PDB ID:3IX3), and PqsR (PDB ID:4JVD) were obtained from the RCSB database. The crystal structure of RhlR has not been published yet; its protein structure (UniProt ID: P54292) was obtained from the UniProt database referring to Chadha’s method (Chadha et al. 2022b). Subsequently, the 3D structures were then optimized by ChemBio3D Ultra1 4.0, PyMOL 4.3.0, and AutodockTools 1.5.6 and saved in the “pdbqt” format for further docking. The search space of AutoDock Vina 1.1.2 was defined with dimensions of size_x: 40, size_y: 40, and size_z: 40 (with a grid point spacing of 0.375 Å), exhaustiveness was set to 8, while other docking parameters were set by default. Then, the docking scores of the protein-small molecule docking combinations were calculated, and the interaction force analysis and 3D and 2D angle visualization were performed using PyMOL 4.3.0 and Discovery Studio 2019. Binding free energy below −5 kcal/mol was considered a relatively stable interaction between ligands and receptors.

### Molecular dynamics simulation

According to the approach of Anju et al., molecular dynamics (MD) simulations were performed to investigate the stability of protein receptors binding with small molecule ligands further (Anju et al. 2021). The Amber force field of the Gromacs 2020.6 simulation package was used. All the complexes were placed in a cubic box with a size of 1.0 Å along with the SPC water model as the solvent. The temperature of the simulation system was maintained at 310 K in a vacuum environment, and the simulation time lasted for 100 ns. Root Mean Square Deviation (RMSD) was used to analyze the relative stability of protein-small molecule binding during the simulation. Root Mean Square Fluctuation (RMSF) shows the structural adaptability of each residue of the protein to analyze the flexibility and motion intensity of amino acid residues. The radius of gyration (Rg) can characterize the compactness of the protein structure and the looseness of the protein peptide chain. Additionally, the stability of the protein-small molecule binding was evaluated by monitoring the formation of H-bond numbers over time.

### Statistical analysis

All data in this study were analyzed using IBM SPSS Statistics 21.0. GraphPad Prism 9.0 and OriginPro 2022 were used for graph plotting. All tests were repeated at least three times independently, and data were expressed as mean ± standard deviation (*M* ± SD). *P* < 0.05 was considered as the level of significance.

## Results

### Favorable interactions exist between quercetin and LasB

**Fig. 1:**
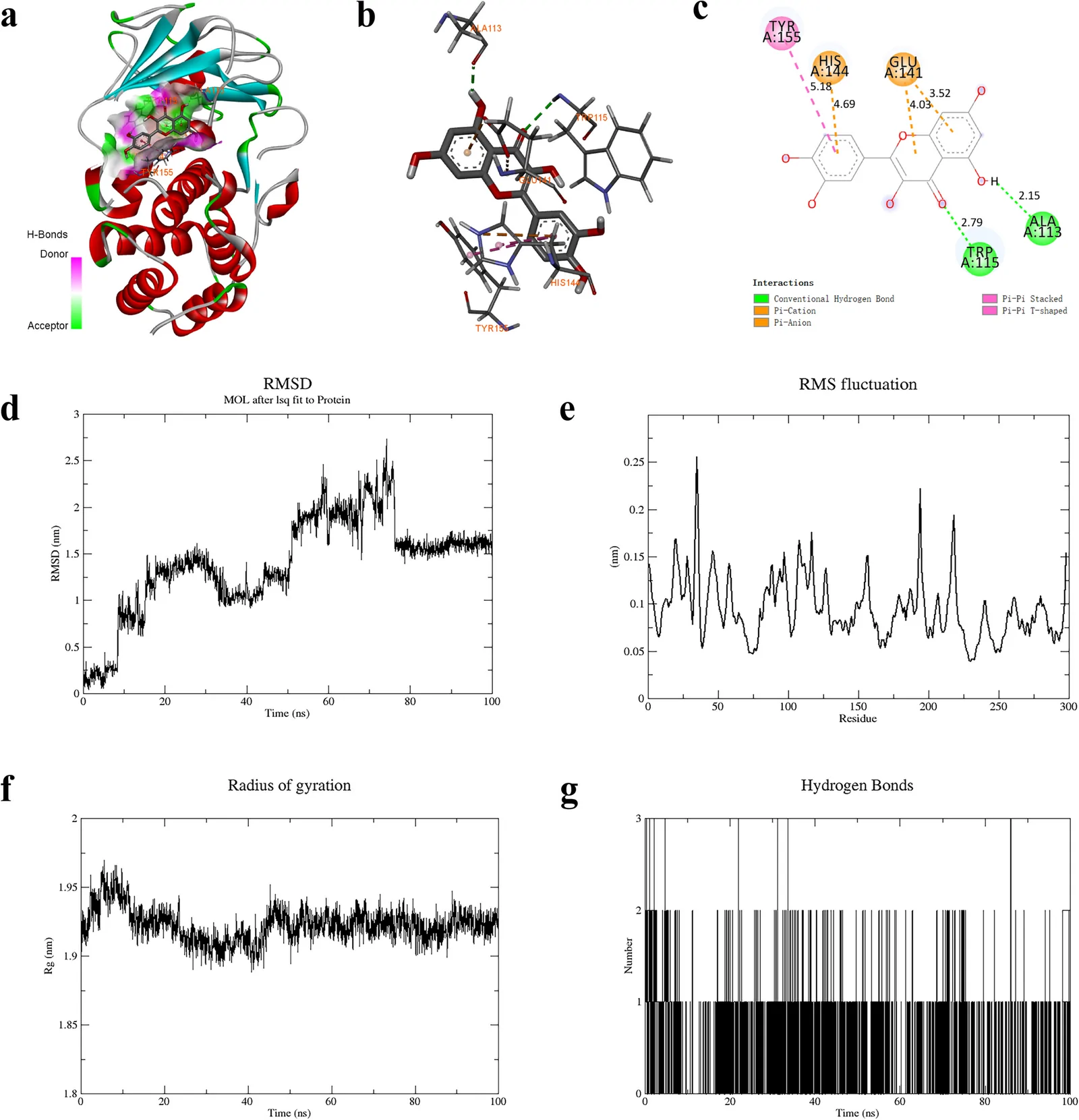
Molecular docking and molecular dynamics simulation of quercetin with LasB. **a** Overall 3D. **b** Local 3D. **c** Local 2D. **d** RMSD. **e** RMSF. **f** Rg. **g** H-bond number. H-bonds produced are in green dashed lines, while VDWs are in pink and brown dashed lines.

To investigate the structural basis of quercetin binding to LasB, the binding energy required was −7.9 kcal/mol, indicating a favorable binding action. The interactions were mainly attributed to the formation of hydrogen bonds (H-bonds) and the Van der Waals hydrophobicity (VDWs). H-bonds were formed with the amino acid residues ALA113 and TRP115 with lengths of 2.15 Å and 2.79 Å, and VDWs were formed with hydrophobic residues GLU141, HIS144, and TYR155 with lengths of 3.52 Å, 4.03 Å, 4.69 Å, and 5.18 Å (Fig. 1a–c). These active sites may have significant implications for the reciprocal binding of quercetin and LasB, which could potentially provide a theoretical foundation for the antivirulence effects of quercetin.

During the MD simulation, the RMSD curve showed the RMS displacement of the small molecule with the protein. Combined with the motion trajectory of the complex, it can be seen that the amplitude was small throughout the simulation. The RMSD curve first rose to vibrate around 1 nm. Subsequently, it rose to vibrate around 2 nm at approximately 50 ns and then plummeted to around 1.5 nm at 80 ns. Afterward, it maintained a smaller range of oscillation until the end of the simulation. The obtained result suggests that the protein and ligand structure reached a dynamically stable conformation at the end of the simulation (Fig. 1d). According to the RMSD results, 20 ns after truncation, the RMSF analysis of the LasB protein amino acids showed that there was a weak jitter in the simulation process, with the amplitude distribution between 0.05 and 0.25 nm. Hence, the RMSF value of the amino acid residues was generally modest, indicating stable ligand binding to the protein (Fig. 1e). The Rg analysis indicated that the overall vibrational amplitude of the LasB protein is small and remained relatively stable in the 1.875–1.975 nm range. This suggests that the protein-ligand complex is relatively stable during the simulation (Fig. 1f). In terms of H-bonds, quercetin and LasB can form up to 3 pairs of H-bonds within 100 ns and 1~2 pairs of H-bonds can be formed most of the time (Fig. 1g). These results indicate that quercetin and LasB can form a stable binding complex.

### Sub-MICs of quercetin exhibit no significant inhibitory effect on the growth of PAO1

**Fig. 2:**
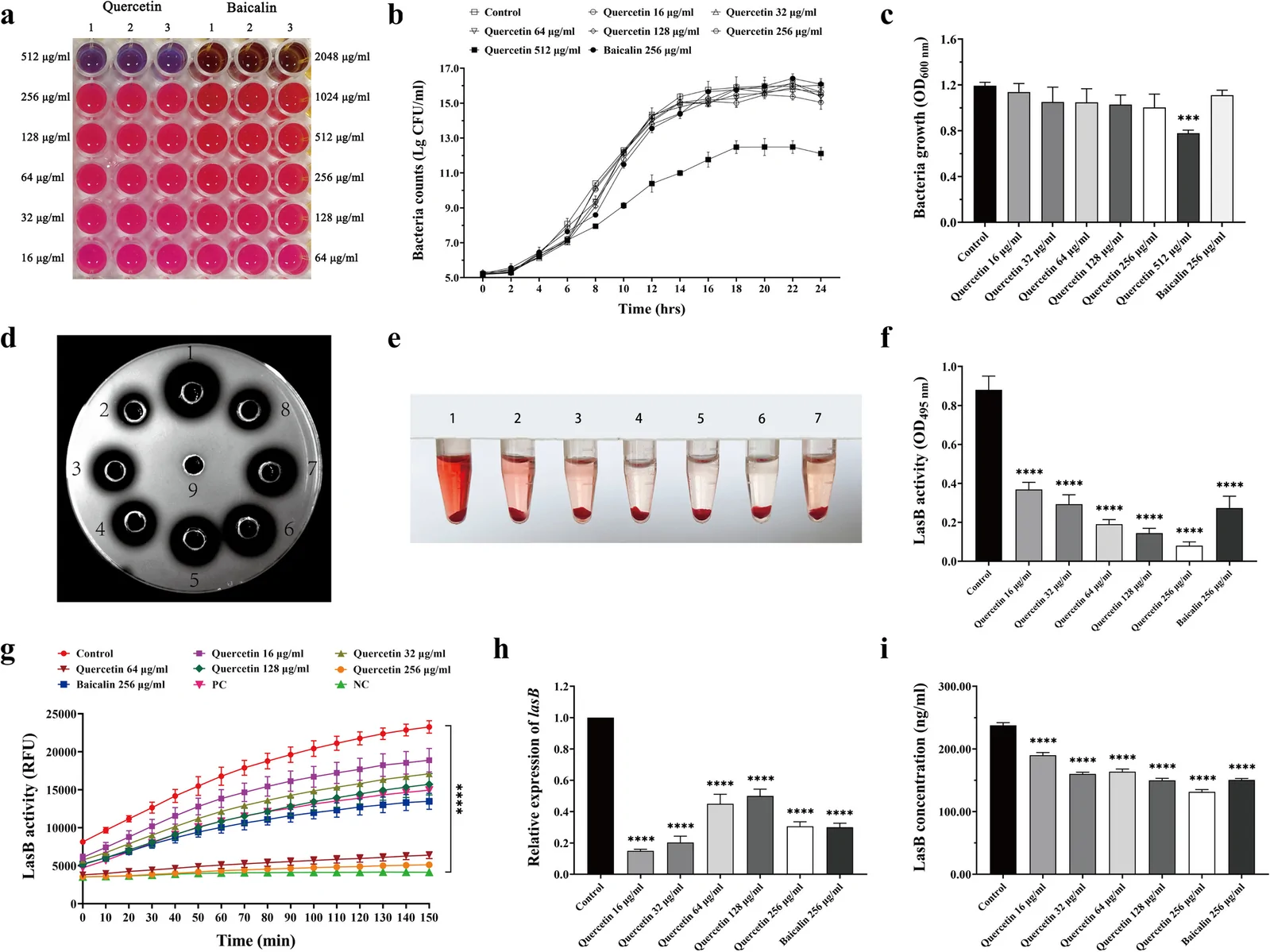
Effects of quercetin on the growth and LasB elastase of *P. aeruginosa* PAO1. **a** MICs of quercetin and baicalin. **b** Bacterial colony counts of PAO1 treated with quercetin or baicalin at the interval of 2 h for a duration of 24 h. **c** OD600 nm of PAO1 treated with quercetin or baicalin after 24 h incubation. **d** Elastin agar assay. 1: control, 20 mm; 2: quercetin 256 μg/ml, 11 mm; 3: quercetin 128 μg/ml, 14 mm; 4: quercetin 64 μg /ml, 14.5 mm; 5: quercetin 32 μg/ml, 15.5mm; 6: quercetin 16 μg/ml, 16 mm; 7: quercetin 16 μg/ml, 16 mm; 8: baicalin 256 μg/ml, 13 mm; 9: LB, no hydrolysis area. mm represents the diameter size of the hydrolyzed area. **e** Elastin-Congo red assay. 1: control; 2: quercetin 16 μg/ml, 11 m; 3: quercetin 32 μg/ml; 4: quercetin 64 μg /ml; 5: quercetin 128 μg/ml; 6: quercetin 256 μg/ml; 7: baicalin 256 μg/ml. **f** OD495 nm of elastin-Congo red assay. **g** Fluorescent-labeled elastin assay. Ex/Em = 485 nm/530 nm. PC, positive control; NC, negative control; RFU, relative fluorescence unit. **h** Relative expression level of *lasB* by RT-qPCR. **i** LasB concentration by ELISA. The data are presented as *M* ± SD of three independent experiments. ***P<0.001, ****P<0.0001 vs. the control group.

The broth microdilution method revealed that the MICs of PAO1 to quercetin and baicalin were 512 μg/ml and 2048 μg/ml, respectively (Fig. 2a). To evaluate quercetin’s antivirulence effect, instead of sterilization or bacteriostasis, the 24 h growth curves were plotted to assess the bacterial survival treated with sub-MICs of quercetin and baicalin (Fig. 2b). No significant differences in the trend bacterial growth or viable bacterial count were observed for PAO1 when treated with 16~256 μg/ml of quercetin or 256 μg/ml of baicalin, as compared to the control group. However, the growth of PAO1 was significantly inhibited when the concentration of quercetin reached 512 μg/ml. Meanwhile, after 24 h of treatment with either quercetin or baicalin, the OD600 of the culture of each group was measured to assess the bacterial density (Fig. 2c). Only the culture treated with quercetin at a concentration of 512 μg/ml presented a significantly lower OD600 than the control group’s (*P* < 0.05). Consequently, the sub-MICs of quercetin were employed in the subsequent study.

### Quercetin inhibits LasB activity and relative expression of *lasB* gene as well as LasB concentration of PAO1

The degrading activity of LasB upon elastin was examined to illustrate the inhibitory effect of quercetin on LasB activity. Results from the elastin agar assay suggest that the maximum inhibition of hydrolysis occurred at 256 μg/ml quercetin. The hydrolysis area gradually increased with the decreasing concentrations of quercetin. Notably, the hydrolysis areas of 128 μg/ml and 64 μg/ml quercetin closely resembled those of 256 μg/ml baicalin and were significantly less than those of the control group. By comparison, the hydrolysis areas of 16 μg/ml quercetin were slightly smaller than those of the control group (Fig. 2d). The ECR assay showed that the control group released the highest amount of Congo red, with a decreasing trend observed as the concentration of quercetin increased (Fig. 2e). The absorbance of the ECR supernatant showed that the LasB activity was most potent in the control group but gradually decreased as the concentration of quercetin increased (*P* < 0.05) (Fig. 2f). Compared to the control group, the elastin degradation activity of LasB was decreased by 57.84±5.42%, 66.24±6.71%, 78.12±3.65%, 83.35±3.37%, 90.81±2.26%, and 68.40±7.85%, respectively, when exposed to 16, 32, 64, 128, and 256 μg/ml of quercetin and 256 μg/ml of baicalin. Quercetin at 256 μg/ml showed the most effective inhibitory effect, while baicalin at 256 μg/ml exhibited a similar effect to quercetin at 32 μg/ml. The fluorescent substrate assay showed an upward trend of RFU among the treated groups in 2.5 h, except for the negative control (Fig. 2g). This indicates that elastin labeled with fluorescence substrates underwent proteolysis by elastase with prolonged reaction time. In addition, the RFU at various sub-MICs of quercetin differed in decrease rates when compared to the control group. In particular, concentrations of 64 μg/ml and 256 μg/ml saw a particular decline (*P*<0.05). The sub-MICs of quercetin could inhibit the hydrolytic activity of LasB elastase secreted by PAO1 throughout the 2.5 h incubation period.

In comparison to the control group, the relative expression levels of the *lasB* gene decreased by 85.11±0.77%, 79.89±2.64%, 54.88±5.35%, 50.26±3.50%, and 69.77±2.34% when exposed to quercetin of 16 μg/ml, 32 μg/ml, 64 μg/ml, 128 μg/ml, and 256 μg/ml, respectively (*P* < 0.05) (Fig. 2h). Additionally, the relative expression level of *lasB* decreased by 70.09±2.39% in the presence of 256 μg/ml baicalin (*P* < 0.05). Interestingly, quercetin displayed stronger inhibitory effects on *lasB* expression at lower concentrations compared to higher ones. The most potent inhibitory effect was observed at 16 μg/ml, suggesting that the inhibitory effects on *lasB* are not influenced by the concentration of quercetin. Fig. 2i illustrates that the concentration of LasB decreased to different extents upon treatment with sub-MICs of quercetin (*P* < 0.05). Furthermore, 256 μg/ml of quercetin exhibited a stronger inhibitory effect at the same concentration compared with that of baicalin, followed by 128 μg/ml of quercetin. Hence, sub-MICs of quercetin effectively suppress the secretion of LasB from PAO1.

### Quercetin suppresses LasB activity and relative expression of *lasB* gene of *P. aeruginosa* clinical isolates

**Fig. 3:**
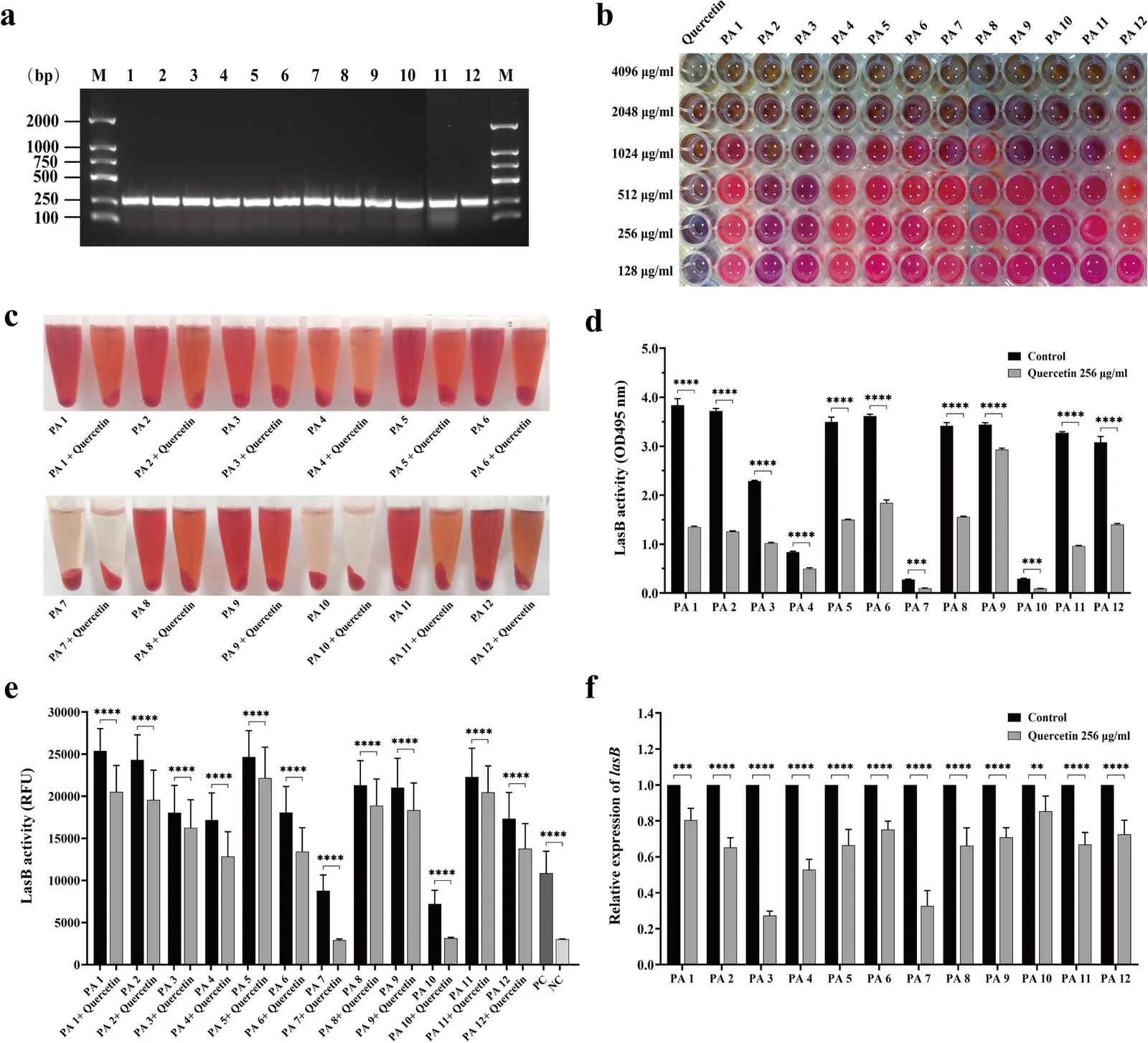
Effects of quercetin on LasB activity and *lasB* gene expression of *P. aeruginosa* clinical isolates. **a** Electrophoretic patterns of *lasB* of PA 1-12. M: DNA marker (100–2000 bp); lanes 1–12: *lasB* of PA 1–12, respectively. **b** MICs of quercetin against PA 1–12. **c** Elastin-Congo red assay. **d** OD495 nm of elastin-Congo red assay. **e** Fluorescent-labeled elastin assay. Ex/Em = 485 nm/530 nm. PC, positive control; NC, negative control; RFU, relative fluorescence unit. **f** Relative expression level of *lasB* by RT-qPCR. The data are presented as *M* ± SD of three independent experiments. **P<0.01, ***P<0.001, ****P<0.0001 vs. the control group.

Given the effect of quercetin on the reference strain PAO1, we randomly selected 12 clinical isolates of *P. aeruginosa* to investigate whether quercetin could be extended to other *P. aeruginosa* strains. Subsequent DNA electrophoresis confirmed the presence of the lasB gene in these 12 *P. aeruginosa* strains (Fig. 3a). The resazurin reduction assay was conducted to evaluate the MICs of these strains and to determine a suitable concentration of quercetin. All clinical isolates had MICs to quercetin that > 512 μg/ml (Fig. 3b). Consequently, a concentration of 256 μg/ml was chosen for subsequent experiments. The elastin-Congo red assay demonstrated variations in LasB activity between different clinical isolates (Fig. 3c, d). Notably, PA 1, PA 2, PA 5, PA 6, PA 8, and PA 9 exhibited higher LasB activities, whereas PA 7 and PA 10 were significantly weaker. After exposure to 256 μg/ml of quercetin, all strains showed a significant decrease in their elastin degradation capacity when compared to the control group (*P* < 0.05). A consistent trend was shown in the elastin fluorescent substrate assay (Fig. 3e), where the fluorescence intensity of each strain considerably decreased when treated with 256 μg/ml of quercetin compared to the control group (*P* < 0.05). Additionally, each strain exhibited a significant reduction in the expression levels of the *lasB* genes (*P* < 0.05), with the most notable decrease observed in PA3 and PA7 (Fig. 3f). Therefore, these results suggest that quercetin is effective in inhibiting the activity of the LasB elastase and the expression of the *lasB* gene in *P. aeruginosa* clinical isolates.

### Significant positive correlations exist between *lasB* gene and QS system regulatory genes in *P. aeruginosa* clinical isolates

**Fig. 4:**
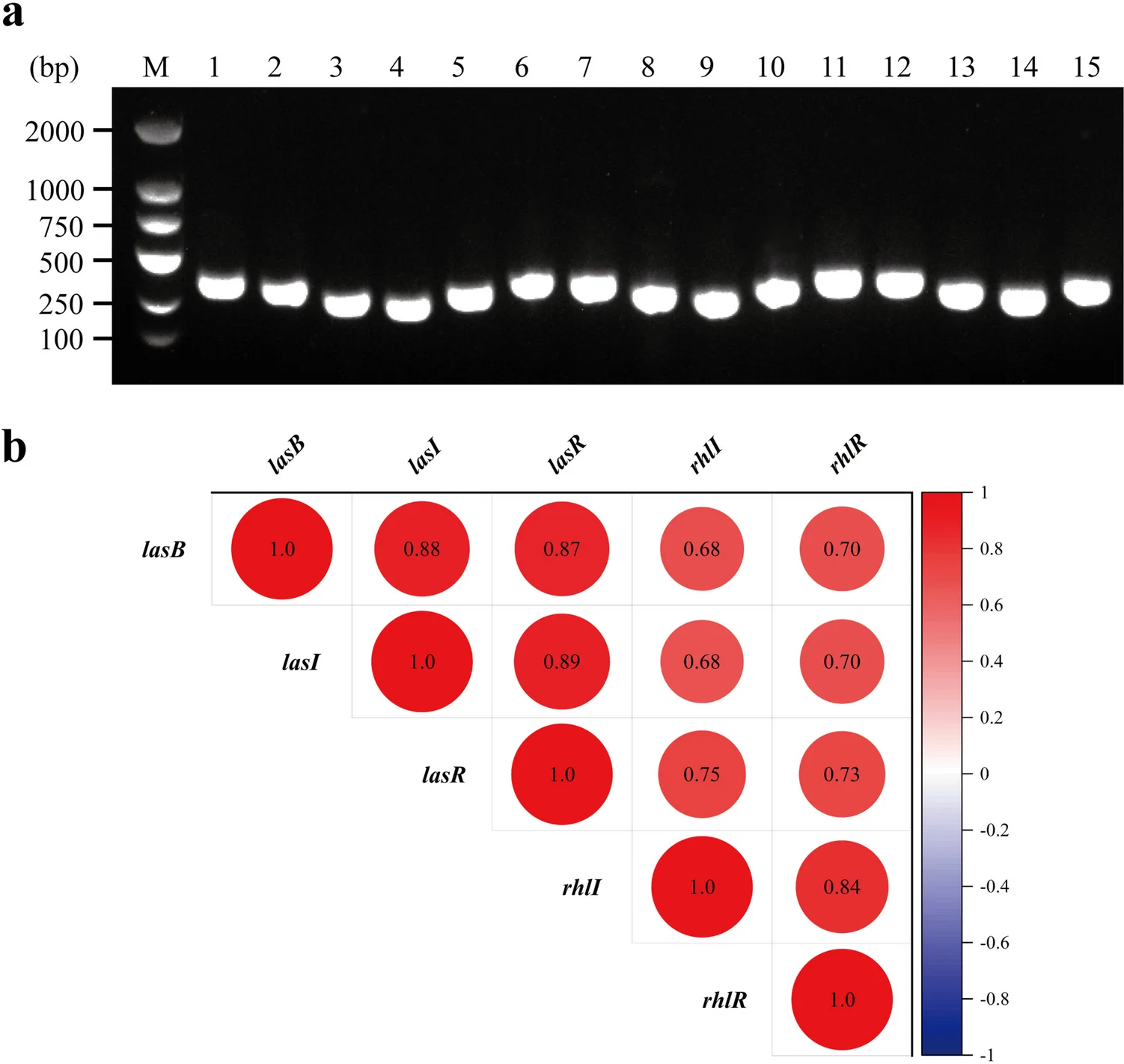
Correlation analysis of *lasB* gene and QS system regulatory genes in clinical isolates. **a** Electrophoretogram of *lasI*, *lasR*, *rhlI*, *rhlR*, and *lasB* gene of *P. aeruginosa* clinical strains. M: DNA marker (100–2000 bp); lanes 1–5, 6–10, and 11–15 represent *lasI*, *lasR*, *rhlI*, *rhlR*, and *lasB* of clinical strains 1, 11, and 22, respectively. **b** Correlation analysis of the relative expression levels of virulence gene *lasB* and QS system genes *lasI*, *lasR*, *rhlI*, and *rhlR* in clinical isolates. |*r*| ≥ 0.8, very highly correlated; 0.6 ≤ |*r*| < 0.8, highly correlated; 0.4 ≤ |*r*| < 0.6, moderately correlated; 0.2 ≤ |*r*| < 0.4, low correlated; |*r*| < 0.2, essentially uncorrelated.

The colony PCR was used to identify the 70 clinical strains of *P. aeruginosa* that simultaneously expressed *lasI*, *lasR*, *rhlI*, *rhlR*, and *lasB* genes (partial results are shown in Fig. 4a). Subsequently, RT-qPCR was carried out to determine the relative expression levels of the genes to assess their correlation. The results indicated a positive correlation between the *lasB* gene and the genes of Las and Rhl systems, specifically *lasI*, *lasR*, *rhlI*, and *rhlR* (*r* = 0.881, 0.868, 0.684, 0.697, *P* < 0.05) (Fig. 4b). It is widely acknowledged that a correlation of |*r*| ≥ 0.8 indicates an extremely high correlation between two variables, whereas 0.6 ≤ |*r*| < 0.8 denotes a high correlation. As such, the findings of this study revealed a strong correlation between the *lasB* gene and both the Las and Rhl systems. Furthermore, the results supported the notion that the Las system functions as the primary regulatory mechanism in the QS system of *P. aeruginosa*. Moreover, there were significant correlations between the QS subsystems of *P. aeruginosa*. Specifically, *lasI* and *lasR* of the Las system (*r* = 0.892) as well as *rhlI* and *rhlR* of the Rhl system (*r* = 0.836) displayed significant strong correlations (*P* < 0.05).

### The LasB activity and *lasB* gene expression exhibit a significant decrease in the *ΔlasI and ΔlasIΔrhlI* mutants of PAO1

**Fig. 5:**
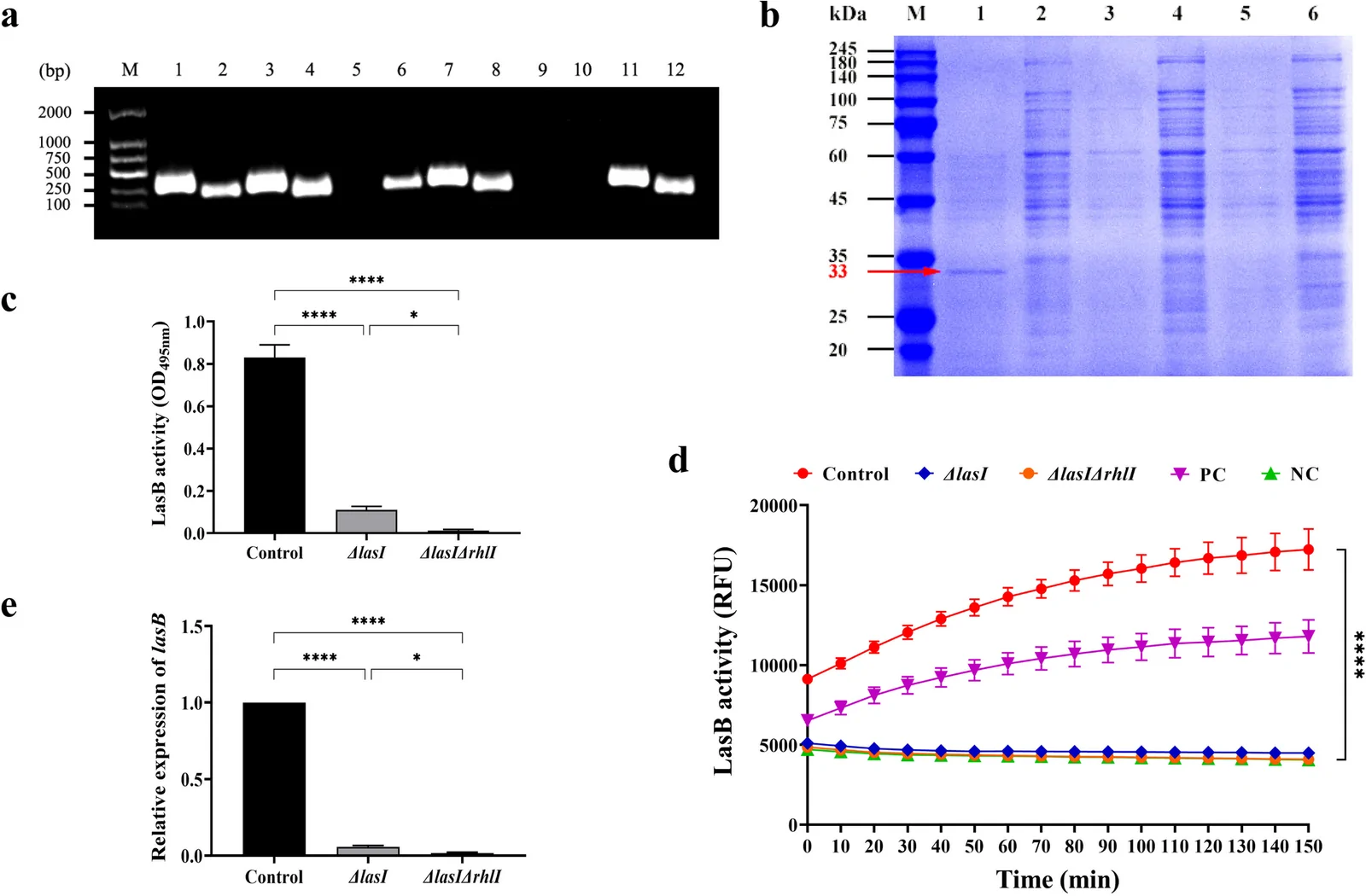
Effects of *ΔlasI* and *ΔlasIΔrhlI* mutant strains of PAO1 on *lasB* gene expression and LasB activity. **a** Electrophoretic patterns of PAO1, *ΔlasI*, and *ΔlasIΔrhlI*. M: DNA marker (100–2000 bp); lanes 1–4, 5–8, and 9–12 represent the expression of *lasI*, *rhlI*, *lasR*, and *rhlR* genes of PAO1, *ΔlasI*, and *ΔlasIΔrhlI* strains, respectively. **b** SDS-PAGE protein electrophoresis. M: protein marker (20–245 kDa); lanes 1, 3, and 5 represent the secretory protein of PAO1, *ΔlasI*, and *ΔlasIΔrhlI* culture supernatant, respectively; lanes 2, 4, and 6 represent the bacterial protein of PAO1, *ΔlasI*, and *ΔlasIΔrhlI* thallus, respectively. **c** Elastin-Congo red assay and **d** Fluorescent-labeled elastin assay were used to determine the LasB activity. **c** Relative expression levels of *lasB* by RT-qPCR. All the data are presented as the *M* ± SD of three independent experiments. *P<0.05, ****P<0.0001 vs. the control group.

The *lasI* and *rhlI* genes encode the self-inducible synthases of the Las and Rhl subsystems, respectively. Using homologous recombination, we obtained the *ΔlasI* and *ΔlasIΔrhlI* mutants of PAO1 (Fig. 5a). SDS-PAGE analysis (Fig. 5b) showed a prominent protein band at 33 kDa in the supernatant secreted by PAO1 (lane 1), indicating the crucial role of LasB elastase in the proteins secreted by PAO1. Interestingly, the absence of a visible band at 33 kDa in *ΔlasI* and *ΔlasIΔrhlI* (lane 3 and lane 5) suggests that the deletion of the *lasI* and *rhlI* genes has a marked inhibitory effect on the secretion of LasB elastase. The elastin Congo red assay (Fig. 5c) revealed a significant reduction in LasB activity in *ΔlasI* and *ΔlasIΔrhlI* compared to PAO1 (*P <* 0.05). Specifically, the *ΔlasI* decreased by 86.74±1.50%, while the *ΔlasIΔrhlI* decreased by 98.51±0.68%. Similarly, in the elastin fluorescence substrate assay (Fig. 5d), the excited fluorescence intensity was consistently absent in the *ΔlasI* and *ΔlasIΔrhlI* supernatants compared with the control (*P* < 0.05) and even at almost the same level as the negative control. RT-qPCR (Fig. 5e) showed that the deletion of the lasI and rhlI genes resulted in a significant decrease in *lasB* gene expression (*P* < 0.05), of which *ΔlasI* demonstrated a downregulation of 94.30±1.36%, and *ΔlasIΔrhlI* displayed a decrease of 98.43±0.92%. Since *lasI* is located upstream of the QS system, it plays a dominant role in the regulation of *lasB* gene expression and LasB elastase production. Additionally, the joint regulation of *lasI* and *rhlI* accounts for the vast majority of *lasB* gene expression and LasB elastase production. Our findings support this theory.

### Quercetin downregulates the relative expression levels of key regulatory genes in the QS system of PAO1

**Fig. 6:**
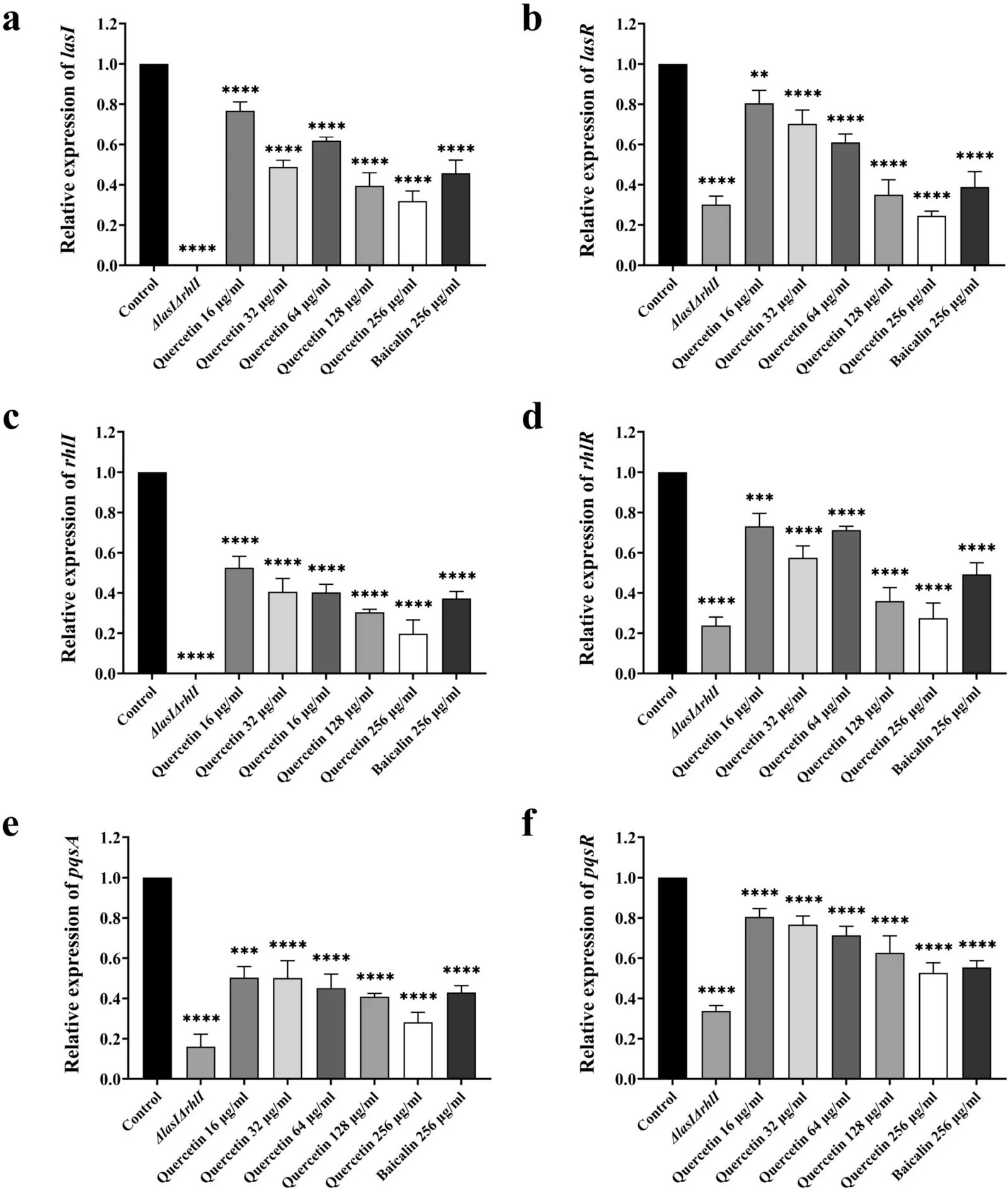
Relative expression levels of *lasI*, *lasR*, *rhlI, rhlR*, *pqsA*, and *pqsR* in the QS system of PAO1 treated with the sub-MICs of quercetin. **a** Relative expression level of *lasI*. **b** Relative expression level of *lasR*. **c** Relative expression level of *rhlI*. **d** Relative expression level of *rhlR*. **d** Relative expression level of *pqsA*. **d** Relative expression level of *pqsR*. All the data are presented as the *M* ± SD of three independent experiments. **P<0.01, ***P<0.001, ****P<0.0001 vs. the control group.

This study investigated how quercetin affects key regulatory genes (*lasI*, *lasR*, *rhlI*, *rhlR*, *pqsA*, and *pqsR*) in the QS system. Compared to the control group, sub-MICs of quercetin (16~256 μg/ml) and baicalin at 256 μg/ml significantly downregulate the transcription levels of *lasI*, *lasR*, *rhlI*, *rhlR*, *pqsA*, and *pqsR* (*P* < 0.05), as shown in Fig. 6. As the concentration of quercetin increased, the QS regulatory genes showed a significant downward trend. Specifically, the *lasI* genes were downregulated by 23.32±3.67%, 51.15±2.78%, 38.10±1.58%, 60.56±5.38%, and 68.07±4.10%, respectively (Fig. 6a). Similarly, *lasR* was downregulated by 19.47±5.21%, 29.76±5.62%, 38.89±3.39%, 64.95±6.07%, and 75.42±1.89% (Fig. 6b). As for Rhl system, the expression of the *rhlI* gene was suppressed by 47.43±4.62%, 59.37±5.36%, 59.75±3.32%, 69.57±1.22%, and 80.29±5.65% (Fig. 6c); the *rhlR* gene was suppressed by 26.89±5.23%, 42.50±4.83%, 28.79±1.58%, 64.10±5.53%, and 72.52±6.20% (Fig. 6d). The *pqsA* gene was downregulated by 49.57±4.43%, 49.85±7.02%, 54.87±5.73%, 59.17±1.42%, and 71.92±4.06%, respectively (Fig. 6e). Finally, the expression level of the *pqsR* was suppressed by 19.45±3.32%, 23.25±3.47%, 28.70±3.73%, 37.33±6.90%, and 47.24±4.06% (Fig. 6f). Interestingly, the treatment of 256 μg/ml baicalin resulted in a decrease of *lasI*, *lasR*, *rhlI*, *rhlR*, *pqsA*, and *pqsR* by 54.26±5.32%, 61.12±6.29%, 62.69±2.82%, 50.78±4.74%, 57.08±2.84%, and 44.58±2.73%, correspondingly. It is evident that at the same concentration, quercetin had a superior inhibitory effect than baicalin. Furthermore, *ΔlasIΔrhlI* resulted in a notable decrease in the expression levels of *lasR*, *rhlR*, *pqsA*, and *pqsR* genes. This decline is related to the regulatory cascade mechanism of the QS system.

### Quercetin inhibits the AHLs in the QS system of PAO1

**Fig. 7:**
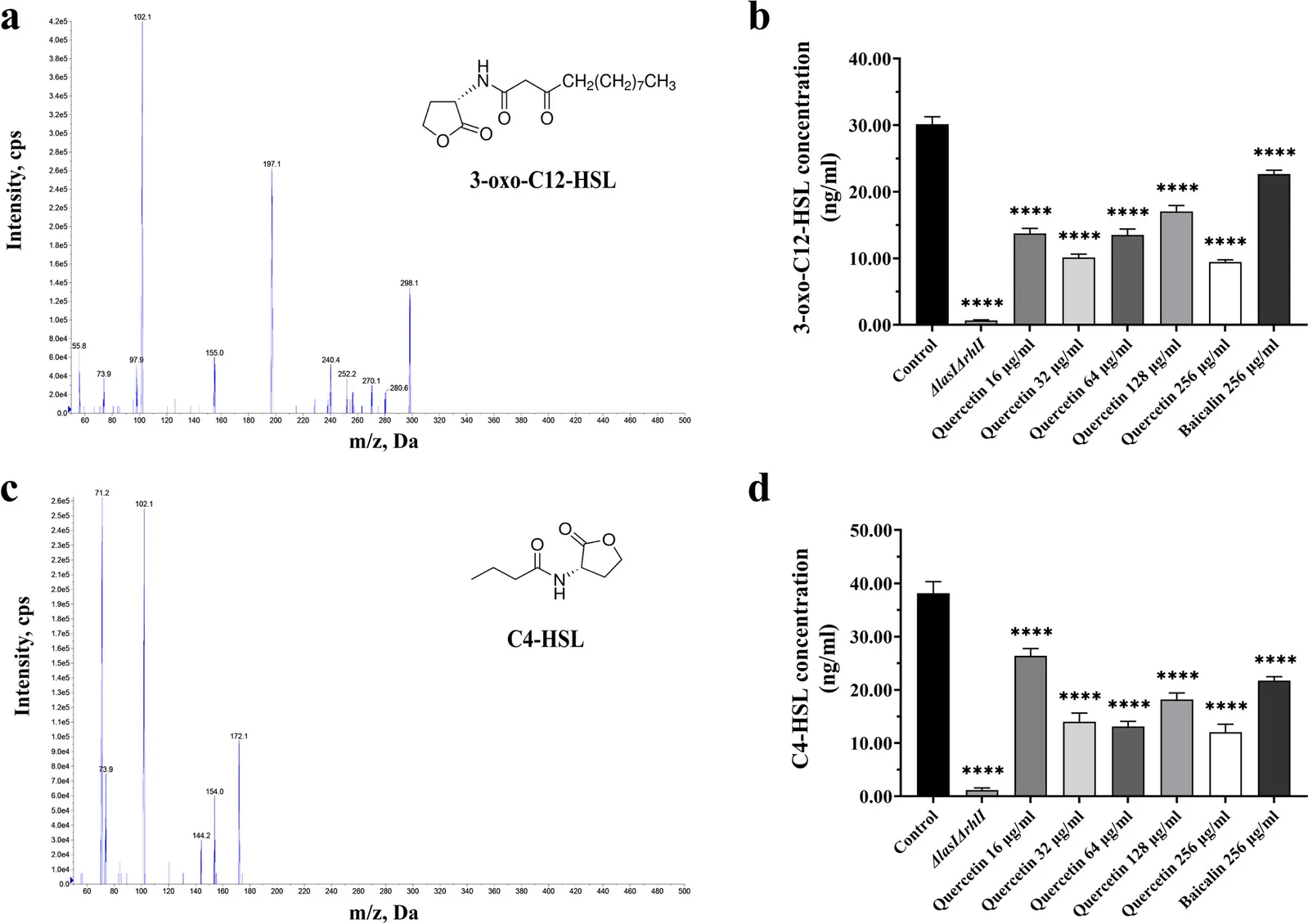
The impacts of quercetin on the synthesis of AHLs extracted from cultural supernatant of PAO1 evaluated by LC-MS/MS. **a** Characteristic fragments originating from the 3-oxo-C12-HSL. **b** Effects of quercetin on the 3-oxo-C12-HSL synthesis of PAO1. **c** Characteristic fragments originating from the C4-HSL. **d** Effects of quercetin on C4-HSL synthesis of PAO1. Blue lines indicate peaks for characteristic fragments of the AHLs. All the data are presented as the *M* ± SD of three independent experiments. ****P<0.0001 vs. the control group.

To evaluate the effects of quercetin on autoinducers in the QS system, AHLs were extracted from cultures of PAO1. The concentrations of 3-oxo-C12-HSL and C4-HSL were quantified by the fragment peaks (daughter ions) separated by Q3. These peaks resulted from the characteristic fragments of parent ions that were separated by Q1 and passed through the Q2 impact chamber. Fig. 7a, c shows the isolated characteristic fragments from the reference standards of 3-oxo-C12-HSL and C4-HSL. Treatment with sub-MICs of quercetin at concentrations of 16 μg/ml, 32 μg/ml, 64 μg/ml, 128 μg/ml, and 256 μg/ml resulted in a significant decrease in the level of 3-oxo-C12-HSL by 54.55±1.58%, 66.51±0.82%, 55.30±2.43%, 43.50±2.09%, and 68.61±0.60%, respectively, compared to the control group (*P* < 0.05). Moreover, the inhibitory effect was more pronounced than that of baicalin at a concentration of 256 μg/ml, demonstrating only an inhibition rate of 24.65±1.74% (Fig. 7b). Meanwhile, the levels of C4-HSL exhibited a similar trend upon exposure to identical concentrations of quercetin, with reductions of 30.85±1.02%, 63.37±2.20%, 65.62±0.58%, 52.32±0.52%, and 68.53±2.17% observed, respectively. In contrast, when exposed to baicalin at a concentration of 256 μg/ml, only a decrease of 42.93±1.91% was registered (*P* < 0.05) (Fig. 7d). The synthesis of 3-oxo-C12-HSL and C4-HSL in *ΔlasIΔrhlI* mutant strain of PAO1 was found to be insufficient. In summary, quercetin may disrupt the expression of QS-mediated LasB by inhibiting the AHL synthesis in PAO1.

### Quercetin competitively binds to receptor proteins of the *P. aeruginosa* QS system

**Fig. 8:**
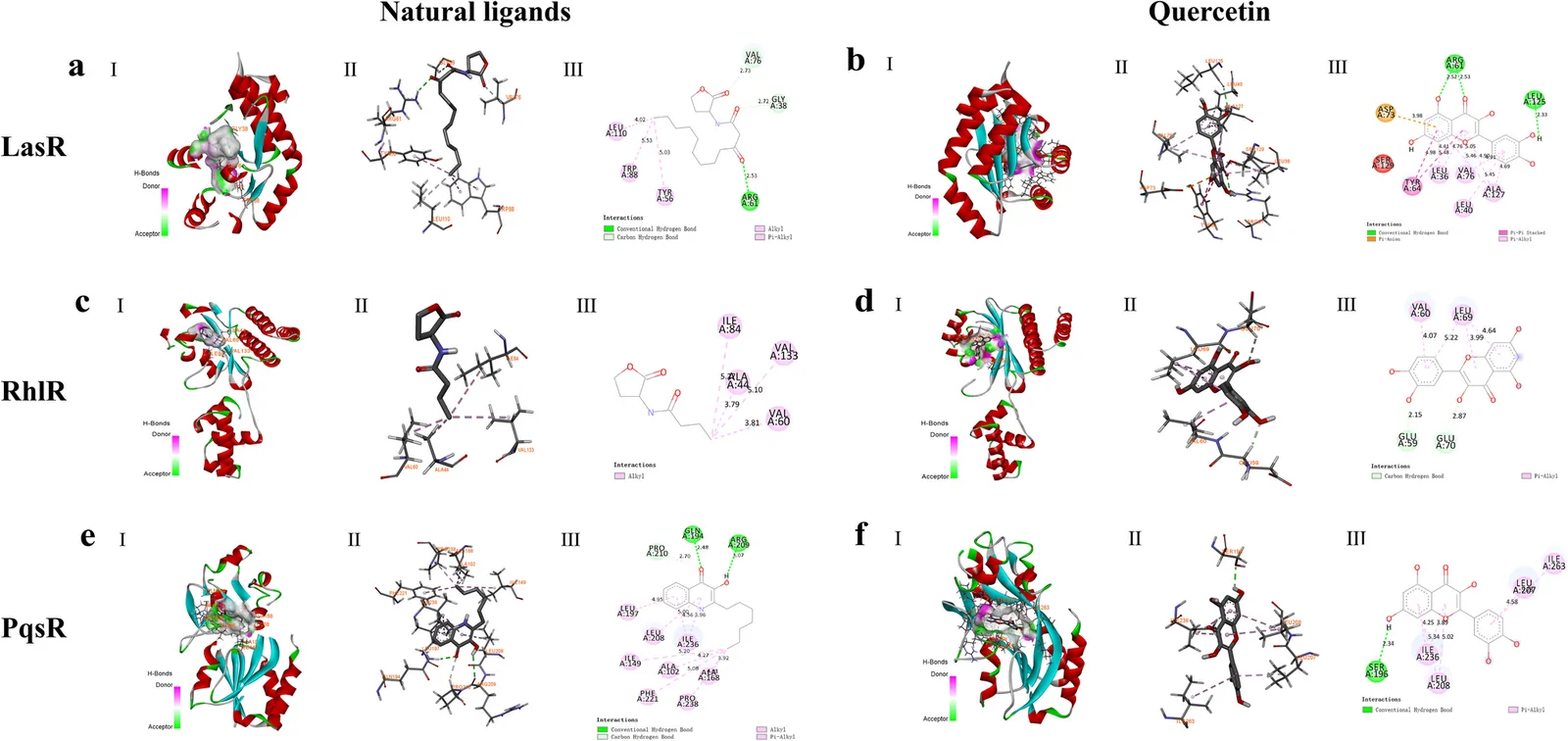
Molecular docking complexes and their interaction modes of small molecules with QS receptor proteins. **a** OdDHL with LasR. **b** Quercetin with LasR. **c** BHL with RhlR. **d** Quercetin with RhlR. **e** PQS with PqsR. **f** Quercetin with PqsR. I, II, and III represent overall 3D, local 3D, and local 2D structure, respectively. H-bonds produced are in green and light green dashed lines and VDWs are in pink, brown, and purple dashed lines.

Given the influence of quercetin on regulatory genes and autoinducer signaling molecules of the PAO1 QS system, molecular docking was used to further explore the structural basis underlying quercetin’s binding to receptor proteins of the QS system. The interaction modes and the docking parameters are shown in Fig. 8 and Table 1. According to the results, natural ligands (Fig. 8a, c, e) and quercetin (Fig. 8b, d, f) can effectively bind to active amino acid residue sites in receptor protein cavities through interaction forces, including H-bonds and VDWs, ultimately forming stable complexes. The lower the docking energy, the more stable the ligand binding to the receptor protein will be. When docking with the LasR receptor protein, OdDHL had a docking energy of −7.8 kcal/mol, while quercetin had −10.2 kcal/mol. Docking with RhlR showed a docking energy of −6.2 kcal/mol for BHL and −7.1 kcal/mol for quercetin. PqsR exhibited a docking energy of −6.9 kcal/mol with PQS compared to quercetin’s requirement of −8.1 kcal/mol. This suggests that quercetin has a higher affinity than natural ligands for binding to LasR, RhlR, and PqsR receptor proteins. Furthermore, regarding quercetin, it formed 3 pairs of H-bonds and 11 pairs of VDWs with LasR to promote stable binding. With RhlR, it formed 2 pairs of H-bonds and 4 pairs of VDWs. When binding to PqsR, there was 1 pair of H-bonds and 6 pairs of VDWs. Compared to the natural ligands, we observed that quercetin not only had lower binding energy with each receptor protein but also shared overlapping interacting amino acid residues with the natural ligand-receptor protein docking complex, as shown in Table 1. These active sites may provide an advantage for quercetin in competing with natural ligands to bind to the receptor proteins, ultimately affecting both the transcription of the virulence gene *lasB* and the production of LasB.

**Table 1:** Docking parameters of small molecules with QS receptor proteins

Ligands	Receptors	Docking energy (kcal/mol)	H-bond residues	VDW residues
OdDHL	LasR	−7.8	**ARG61**, VAL76, GLY38	LEU110, TRP88, TYR56
Quercetin	LasR	−10.2	**ARG61**, LEU125	TYR64, LEU36, VAL76, ALA127, LEU40, ASP73
BHL	RhlR	−6.2	–	ILE84, ALA44, VAL133, **VAL60**
Quercetin	RhlR	−7.1	GLU59, GLU70	LEU69, **VAL60**
PQS	PqsR	−6.9	GLN194, ARG209, PR0210	LEU197, **LEU208**, **ILE236**, ILE149, ALA102, ALA168, PHE221, PRO238
Quercetin	PqsR	−8.1	SER196	LEU207, **LEU208**, **ILE236**, ILE263

Bold highlights amino acid residues that serve as shared binding sites for both quercetin and natural ligands bound to QS receptor proteins

### Quercetin stably binds to receptor proteins of the *P. aeruginosa* QS system

**Fig. 9:**
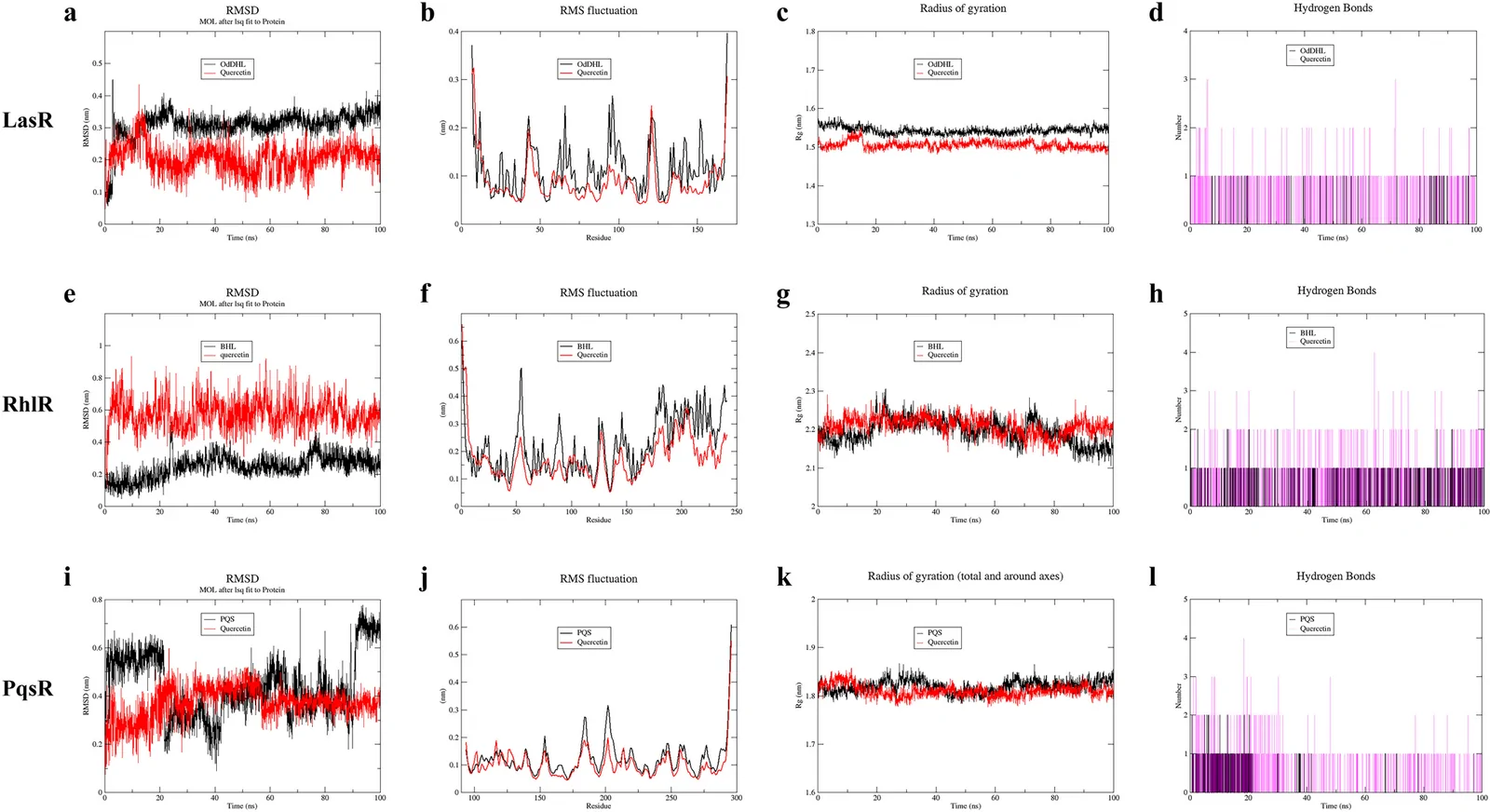
Molecular dynamics simulation of small molecule binding to QS receptor proteins. **a** RMSD of OdDHL/quercetin binding to LasR. **b** RMSF of OdDHL/quercetin binding to LasR. **c** Rg of OdDHL/quercetin binding to LasR. **d** H-bond number of OdDHL/quercetin binding to LasR. **e** RMSD of BHL/quercetin binding to RhlR. **f** RMSF of BHL/quercetin binding to RhlR. **g** Rg of BHL/quercetin binding to RhlR. **h** H-bond number of BHL/quercetin binding to RhlR. **i** RMSD of PQS/quercetin binding to PqsR. **j** RMSF of PQS/quercetin binding to PqsR. **k** Rg of PQS/quercetin binding to PqsR. **l** H-bond number of PQS/quercetin binding to PqsR.

According to the molecular docking results, we further conducted MD simulations to study the dynamics and stability of the docking complexes. During the dynamic simulation between OdDHL/quercetin and LasR, the RMSD curves showed a similar trend. The quercetin complex had a consistently lower RMSD value (approximately 0.2 nm) compared to the OdDHL complex (approximately 0.3 nm) after stabilization, indicating that the quercetin complex structure is stronger than the OdDHL composite structure (Fig. 9a). The RMSF value of the quercetin complex was generally lower than the OdDHL complex, suggesting less fluctuation and greater stability in amino acid residues compared to the OdDHL complex (Fig. 9b). The Rg value of quercetin (approximately 1.5 nm) was lower than that of OdDHL (approximately 1.55 nm), and their changes during the simulation were small, indicating that both reach a stable structural state (Fig. 9c). Moreover, OdDHL and LasR could form a maximum of one H-bond, and usually no H-bond is formed. On the other hand, quercetin could form up to 3 H-bonds, and most of the time 1~2 H-bonds are formed (Fig. 9d). These findings suggest that quercetin binds to LasR receptor protein more firmly compared to OdDHL.

Regarding BHL/quercetin binding with RhlR, the RMSD value exhibited some fluctuations when combined with BHL but remained consistently oscillated around 0.6 nm and maintained a stable trend within 100 ns when combined with quercetin. (Fig. 9e). The RMSF value of the quercetin complex was lower than that of the BHL complex, indicating stronger stability and reduced jitter in amino acid residues (Fig. 9f). Both complexes had similar Rg values, approximately 2.25 nm. However, BHL exhibited slight oscillation and decreased in the last 20 ns, suggesting that a longer simulation may be needed. When combined with quercetin, the Rg remains dynamically stable, indicating stability in their combination (Fig. 9g). The formation of up to 2 pairs of H-bonds between BHL and RhlR was rare, while quercetin could consistently form up to 2 pairs and even up to 4 pairs (Fig. 9h). These results indicate quercetin binds more tightly to LasR than OdDHL does.

When PQS/quercetin was combined with PqsR, the amplitude of the PQS complex varied significantly, while the RMSD value of the quercetin complex oscillated around 0.4 nm with a slight tendency and reached stability after 60 ns (Fig. 9i). The RMSF value of the quercetin complex was lower than that of the PQS complex, indicating higher stability and reduced jitter in amino acid residues (Fig. 9j). The Rg values for binding to the two ligands were generally similar, but PQS exhibited slight oscillations while quercetin maintained dynamic stability, and quercetin showed a slightly lower Rg than PQS after 20 ns (Fig. 9k). In terms of H-bonds, PQS and PqsR could only form up to 2 pairs momentarily, while quercetin could form up to 4 pairs and typically forms 1~2 pairs (Fig. 9l). These findings show greater stability in the binding of quercetin to PqsR.

## Discussions

The resistance of pathogens to antibiotics has become an increasingly prominent public health issue (Huemer et al. 2020). *P. aeruginosa*, an opportunistic pathogen extensively distributed in various environments, is highly prone to acquiring resistance genes. This leads to the rapid transmission of drug resistance and poses a major threat to the treatment of community-acquired and nosocomial infections (Liao et al. 2022). Traditional antimicrobial therapies work by inhibiting bacterial growth or directly killing bacteria, resulting in a surge of MDR and XDR (Chadha et al. 2021). Virulence factors secreted by *P. aeruginosa* can synergistically contribute to the damage of host cells, thereby exacerbating infection and accelerating disease progression (Balasubramanian et al. 2013). Currently, antivirulence therapy is being considered as an alternative when antibiotic treatment proves ineffective. The antivirulence agents do not directly eliminate the bacteria, thus mitigating the selective pressure for resistance development. Instead, they selectively target non-essential pathways to impede virulence factors that are pivotal in establishing infection and inducing disease (Ahmed et al. 2019). Antibiotic alternatives that specifically target bacterial virulence significantly reduce the selective pressure exerted by classical antimicrobials on bacterial growth and contribute to mitigating horizontal gene transfer of drug-resistant genetic elements (Dickey et al. 2017; Totsika 2017). Consequently, it may play a preventive or retarding role in the rapid progression of drug-resistant phenotypes.

LasB, a virulence factor that is capable of cleaving multiple protein substrates in host tissues and the extracellular matrix, is an extracellular protease secreted by *P. aeruginosa* during host infection or culture *in vitro*. Previous studies have shown that LasB plays an important role in hydrolyzing the internal peptide bonds of proteins, particularly the amidogen of hydrophobic amino acid residues (Carson et al. 2012). Additionally, it can degrade immune globulins, complement factors and cytokines, etc., which are involved in manipulating the host immune system to promote *P. aeruginosa* colonization and successful transmission (Santajit et al. 2021).

Chinese herbs and plant-derived compounds have been shown to possess antimicrobial activity, as well as the ability to inhibit bacterial QS systems and virulence factors at low concentrations (Ahmed et al. 2019; Aldawsari et al. 2021; Xu et al. 2019). As a dietary flavonoid compound widely found in plants, quercetin is considered to have antioxidant, anti-inflammatory, antimicrobial, and antivirulence effects (Li et al. 2016). Previous research has demonstrated that quercetin at a concentration of 16 μg/ml can inhibit the biofilm, virulence factors, and QS regulatory genes of *P. aeruginosa* (Ouyang et al. 2016). Further studies have reported that quercetin can modulate *P. aeruginosa* biofilm formation by inhibiting the Las system (Gopu et al. 2015; Ouyang et al. 2020). However, the precise mechanism by which quercetin affects LasB remains unclear. Therefore, our investigation aimed to determine whether quercetin can inhibit LasB via the QS system. To evaluate the antivirulence potential of quercetin against LasB, the structural basis of quercetin/LasB interaction was first predicted by molecular docking. The binding energy of quercetin docked to LasB was −7.9 kcal/mol, indicating a strong affinity activity of quercetin to LasB. These results suggest that the hydrophobic backbone of quercetin can stably interact with the active sites of LasB. As shown in the docking complex, their interactions were mainly achieved through H-bonds and VDWs, which further confirmed our speculations (Fig. 1a–c). MD further simulated the dynamics and stability of the quercetin-LasB binding complex. The RMSD value indicated that the dynamic stability of quercetin-LasB was achieved during the last 20 ns, and there was a relatively small fluctuation in the amplitude of RMSF during this period (Fig. 1d, e). Rg remained relatively constant, and 1~3 H-bonds can be formed in 100 ns (Fig. 1f, g). These results provided evidence for the formation of a stable complex between quercetin and LasB.

In the subsequent *in vitro* experiments, we determined that the MIC of quercetin for PAO1 was up to 512 μg/ml (Fig. 2a). The time-kill assay demonstrated that the treatment groups with sub-MICs of quercetin (16~256 μg/ml) did not show significant direct bactericidal effects in comparison to the control group (Fig. 2b, c). Therefore, sub-MICs of quercetin were used to validate its effect on the LasB elastase of PAO1. To obtain more valuable results, we employed three methods simultaneously to detect LasB activity. To assess the ability of LasB in PAO1 culture supernatant to degrade elastin substrates, the elastin agar plate experiment (Fig. 2d) demonstrated a gradual decrease in the elastin hydrolysis cycle with increasing quercetin concentration (*P* < 0.05). The ECR assay (Fig. 2e, f) indicated a decrease in the release of elastin coupled Congo red with an increasing quercetin concentration (*P* < 0.05). Using elastin labeled with fluorescent substrates, the fluorescence intensity was detected continuously for 150 min (Fig. 2g), and the excited fluorescence intensity was significantly inhibited by quercetin (*P* < 0.05). Strikingly, the LasB activity was even decreased by more than 90% upon exposure to 256 μg/ml quercetin, which exhibited superior inhibitory potency compared to 256 μg/ml baicalin. Taken together, these results indicate that sub-MICs of quercetin have a significant advantage in diminishing LasB activity in the PAO1 culture supernatant and thus exhibiting enormous potential in terms of suppressing the LasB virulence in *P. aeruginosa*. Furthermore, the *lasB* gene expression level and LasB elastase concentration were also significantly reduced compared to the control group (*P* < 0.05), as shown in Fig. 2h, i. Interestingly, the expression level of the *lasB* gene did not exhibit a consistent trend with the observed decrease in LasB activity. Notably, the *lasB* was found to be significantly lower at a concentration of 16 μg/ml quercetin, suggesting that the *lasB* gene may be more sensitive to lower concentrations of quercetin.

Given the significant effect of quercetin on LasB of the *P. aeruginosa* standard strain PAO1, we proceeded to randomly select 12 clinical *P. aeruginosa* strains with different sample sources and drug resistance to determine the universality of quercetin against LasB of *P. aeruginosa*. All these strains were verified to express the *lasB* gene (Fig. 3a). The broth microdilution method showed that the MICs of quercetin varied among different clinical strains, but in general, they were all > 512 μg/ml (Fig. 3b). Consequently, 256 μg/ml quercetin was used for the clinical sample analysis. After 18 h of interaction with ECR, the culture supernatant of each clinical strain showed various shades of red (Fig. 3c), indicating that LasB elastase secreted by different clinical strains exhibited diverse elastin-degrading activities. We believe that LasB is associated with the pathogenicity of clinical samples, and quercetin could potentially function as an antivirulence agent against *P. aeruginosa* provided it proves to exhibit inhibitory effects on LasB. The experimental results were consistent with our expectation that all clinical samples exposed to 256 μg/ml quercetin showed a significantly brighter red shade compared to the untreated control group (Fig. 3c), indicating that quercetin effectively inhibited LasB activity in all 12 clinical samples. These differences were quantified by measuring the absorbance at OD495 nm (Fig. 3d), which showed that regardless of the level of LasB activity in the control group, it could be remarkably reduced by 256 μg/ml quercetin (*P* < 0.05). Similarly, fluorescent-labeled elastin assay demonstrated a noticeable increase in RFU when exposed to *P. aeruginosa* culture supernatants during 150 min. However, treatment with 256 μg/ml of quercetin resulted in a substantial reduction in RFU levels for each sample (*P* < 0.05) (Fig. 3e). The obtained outcome is generally in line with the results of the ECR assay. The relative expression levels of the *lasB* gene in each sample were also significantly downregulated by the intervention of quercetin (*P* < 0.05) (Fig. 3f). The findings further validate the inhibitory effect of quercetin on LasB in clinical strains of *P. aeruginosa*.

The QS system is a highly complex but extensively studied intercellular interaction mode of *P. aeruginosa* (Abisado et al. 2018). It is a genetic regulatory mechanism in bacteria that facilitates intercellular communication and governs the expression of virulence genes based on population density (García-Contreras 2016). Inhibition of QS system has been proposed as a prospective antimicrobial strategy to attenuate the virulence of *P. aeruginosa* (Elfaky et al. 2023; Kumar et al. 2021). This process is mediated by a series of autoinducers and their corresponding transcriptional regulatory proteins (Moura-Alves et al. 2019; Rutherford and Bassler 2012). When an autoinducer binds to a transcriptional regulator to form a complex, it functions as a transcriptional activator, triggering the transcription of target genes (Defoirdt 2018). LasB is a classical Zn^2+^-dependent metalloprotease encoded by the *lasB* gene which is regulated by the QS system. The *lasB* gene is co-regulated by the Las, Rhl, and Pqs subsystems, of which the Las system is dominant and partially regulates the other two sub-systems (Everett and Davies 2021). *lasI*, *rhlI*, and *pqsABCDE* are responsible for the production of the autoinducer synthetases LasI, RhlI, and PqsABCDE. *lasR*, *rhlR*, and *pqsR* are involved in the synthesis of the transcriptional regulators, LasR, RhlR, and PqsR (El-Mowafy et al. 2014).

In this study, to further understand how quercetin acts on the LasB of *P. aeruginosa*, it is imperative for us to identify the resource of LasB. Given the fundamental role of the QS system in the expression of the virulence genes, we speculated that the significant decrease of the *lasB* gene in *P. aeruginosa* PAO1 and clinical isolates might be related to the regulation of the QS system. Therefore, we collected 70 clinical strains of *P. aeruginosa* that expressed both *lasB* and QS system regulatory genes *lasI*, *lasR*, *rhlI*, and *rhlR* (Fig. 4a) and then analyzed the correlations between their gene expression levels, which verified that *lasB* was of positive correlations with QS regulatory genes significantly (*P* < 0.05) (Fig. 4b), and the Las system showed a stronger positive correlation with *lasB* than Rhl system (*P* < 0.05), which was consistent with the previous study (Le Berre et al. 2008). In addition, there was also a significant positive correlation between the Las and Rhl systems, further confirming that the QS system in *P. aeruginosa* was a cascading regulatory network (Lee and Zhang 2015).

By employing homologous recombination, we successfully obtained the single-gene deletion mutant *ΔlasI* and the double-gene deletion mutant *ΔlasIΔrhlI* of PAO1, which are responsible for encoding the Las and Rhl autoinducer synthases of the QS system, respectively. The QS gene profiles of *ΔlasI* and *ΔlasIΔrhlI* were validated (Fig. 5a). The protein gel electrophoresis profiles revealed that LasB is one of the most conspicuous proteins secreted by PAO1 into extracellular space. Notably, when the *lasI* and *rhlI* genes were deleted, the LasB protein bands became undetectable, further confirming the involvement of the Las and Rhl systems in the regulation of LasB (Fig. 5b). As expected, the deletion of the *lasI* and *rhlI* genes significantly reduced LasB activity (*P* < 0.05) (Fig. 5c, d) and *lasB* gene expression (*P* < 0.05) (Fig. 5e), and the deletion of both genes even resulted in the absence of the *lasB* gene and LasB activity. This further confirms that LasB synthesis is closely related to the QS system, in which the Las system and Rhl systems play an important role. Hence, when the regulatory genes in the Las and Rhl systems are absent or mutated, the signaling molecules become inactive or their activity is lowered, leading to a decrease in LasB activity and production, ultimately compromising its virulence impact.

To uncover the mechanism behind quercetin’s effect on LasB, we investigated the effect of quercetin on the QS system of PAO1. Interfering with the QS system of *P. aeruginosa* helps to treat infections without hindering the growth of bacteria (Anju et al. 2021). The effects of quercetin on key regulatory genes and signaling molecules of the QS system were investigated using RT-qPCR and LC-MS/MS. Additionally, molecular docking and MD simulation were utilized to simulate the interaction between quercetin and the transcriptional regulatory proteins to elucidate the effects of quercetin on the QS system. Our experimental findings demonstrate that after sub-MICs of quercetin treatment, the transcript levels of *lasI*, *lasR*, *rhlI*, *rhlR*, *pqsA*, and *pqsR* genes, which encode pivotal autoinducer synthases and transcriptional regulators involved in the QS system, were significantly reduced (*P* < 0.05) (Fig. 6). The *ΔlasIΔrhlI* mutant was employed as a negative control to evaluate the gene expression. Overall, the concentration-dependent effects of quercetin on the tested genes were found to be diverse. Specifically, at a concentration of 256 μg/ml, quercetin exhibited superior inhibitory effects compared to the positive control baicalin. Furthermore, at a lower concentration of 16 μg/ml, each gene demonstrated significant downregulation.

The sub-MICs of quercetin can interfere with the QS system by inhibiting the expression of the *lasI*, *lasR*, *rhlI*, *rhlR*, *pqsA*, and *pqsR* genes in the QS system. The *lasI* and *rhlI* genes control the synthesis of signal molecules C4-HSL and 3-oxo-C12-HSL. Therefore, the degradation of signaling molecules or targe the interaction of signaling molecules with their cognate receptors contributes to the suppression of the QS system (Kumar et al. 2021). Quantitative analysis of signal molecules revealed that the production of C4-HSL and 3-oxo-C12-HSL was significantly suppressed after treatment with quercetin (*P* < 0.05) (Fig. 7). The effect of the QS system on LasB largely depends on the formation of signaling molecules and their associated receptor protein complexes. Therefore, decreasing the AHL signaling molecules results in the direct inhibition of the Las and Rhl system pathways.

Moreover, computational analysis was used to investigate whether quercetin competes for binding with receptor proteins of the QS system. Interactions between compounds depend on the specific groups to which they bind and are responsible for changes in the conformation of the receptor protein (Gopu et al. 2015). According to the molecular docking results, the quercetin’s binding scores with the receptor proteins LasR, RhlR, and PqsR were lower than those of the natural ligands OdDHL, BHL, and PQS (Table 1). Besides, quercetin shared same amino acid interaction residues with natural ligands (Fig. 8). Therefore, quercetin may have advantages over natural ligands in occupying these common active sites of receptor proteins and forming more stable interactions with them. To confirm our findings, we applied MD simulations to analyze RMSD, RMSF, Rg, and H-bond number formed between the docking complexes. We made a comprehensive judgment by combining the values, amplitudes, and overall variation trends of RMSD, RMSF, and Rg, as well as the H-bond numbers. The results further illustrated that quercetin could form more stable complexes with LasR, RhlR, and PqsR receptor proteins during the simulation compared to the natural ligands (Fig. 9). These findings validate that quercetin can act on QS regulatory genes and signaling molecules, competing with natural ligands to bind receptor proteins and form more stable complexes, eventually coordinating the QS system to perform a regulatory function.

In conclusion, the present study demonstrates that quercetin can effectively suppress the production and activity of LasB elastase of *P. aeruginosa in vitro* by interfering with its QS system. Through inhibiting the expression levels of regulatory genes, reducing the synthesis of AHLs, and competing with natural autoinducers to form more stable complexes with receptor proteins, quercetin can exert its potent inhibition on LasB virulence. Therefore, quercetin shows promise as an antivirulence agent against LasB of *P. aeruginosa*. In future research, our focus will be on investigating the preventive and therapeutic effects of quercetin on LasB elastase-induced damage and inflammation *in vitro* and *in vivo* from a pathological perspective. Additionally, we will also explore the effectiveness and feasibility of combining quercetin with conventional antibiotics for the antivirulence therapy of *P. aeruginosa* infections.

## Supplementary information

ESM 1 (PDF 135 kb)

## Data Availability

All data generated or analyzed during this study are included in the submitted manuscript.
