# Supplementary material for: Quercetin: a promising virulence inhibitor of Pseudomonas aeruginosa LasB in vitro
Source: Appl Microbiol Biotechnol. 2024 Jan 5;108:57. doi: 10.1007/s00253-023-12890-w (PMC10770215; doi:10.1007/s00253-023-12890-w)
Supplement: Supplementary file 1 [file 253_2023_12890_MOESM1_ESM.pdf]

Applied Microbiology and Biotechnology

Quercetin: a promising virulence inhibitor of *Pseudomonas aeruginosa* LasB *in vitro*

Yanying Ren[a], Rui Zhu[a,b], Xiaojuan You[a,b], Dengzhou Li[a,b], Mengyu Guo[a], Bing Fei[a], Ying Liu[a], Ximing Yang[f]*, Xinwei Liu[a,b]*, Yongwei Li[a,b,c,d,e]*

a *Henan University of Chinese Medicine, Zhengzhou, 450046, China*

b *Henan Province Hospital of Traditional Chinese Medicine, The Second Affiliated Hospital of Henan University of Chinese Medicine, Zhengzhou, 450002, China*

c *The Key Laboratory of Pathogenic Microbes &Antimicrobial Resistance Surveillance of Zhengzhou, Zhengzhou, 450002, China*

d *Henan Engineering Research Center for Identification of Pathogenic Microbes, Zhengzhou, 450002, China*

e *Henan Provincial Key Laboratory of Antibiotics-Resistant Bacterial Infection Prevention & Therapy with Traditional Chinese Medicine, Zhengzhou, 450002, China*

f *Dongzhimen Hospital of Beijing University of Chinese Medicine, Peking, 100700, China*

**** Corresponding author***

Ximing Yang
Dongzhimen Hospital of Beijing University of Chinese Medicine
No. 5, Haiyuncang, Dongcheng District, Beijing, 100700, China
E-mail: yximing2005@163.com.

XinWei Liu
Henan Province Hospital of Traditional Chinese Medicine
No.6 Dongfeng Road, Jinshui District, Zhengzhou, 450002, China
E-mail: 43154727@qq.com

Yongwei Li
Henan University of Chinese Medicine
No. 156 Jinshui East Road, Chung Dong New Area, Zhengzhou, 450046, China
E-mail: lyw@hactcm.edu.cn

Table S1 Primer sequences used for constructing gene-deficient mutants

Primers	Direction	Sequences (5'-3')
lasI-5F	Forward	CATCTACCAGACGCGAAAGCAGCAC
lasI-5R	Reverse	TACGATCATCTTCACTTCCTCCAAATAGGAAGCTG
lasI-3F	Forward	GTTTCATGACGGGGACCTGTCGGC
lasI-3R	Reverse	CTCAACGATAGCCAGGACTGGCACG
lasI-GmF	Forward	CAGCTTCCTATTTGGAGGAAGTGAAGATGATCGTA CATATGAATATCCTCCTTAGTTCCTATTC
lasI-GmR	Reverse	GCCGACAGGTCCCCGTCATGAAACGAGCTGCTTC GAAGTTCCTA
rhlI-5F	Forward	GAAGTCGAAGGGTTGCTGCGGATGC
rhlI-5R	Reverse	GTGTGAGGTCGTCAGCCGTTTCGC
rhlI-3F	Forward	TTCGATCATGACCAAGTCCCCGTGTCG
rhlI-3R	Reverse	GCTTCGATTACTACGCCTATGGCGTGC
rhlI-AprF	Forward	GCGAAACGGCTGACGACCTCACACGAGCTGCTTC GAAGTTCCTA
rhlI-AprF	Reverse	CGACACGGGGACTTGGTCATGATCGAACATATGAA TATCCTCCTTAGTTCCTATTC

Tables S2 Primer sequences used for conventional PCR

Genes	Direction	Sequences (5’-3’)	Size of product (bp)
lasB	Forward	CAGCACCGACGACAGCAAGAC	274
	Reverse	CAGCGACACCAGCGGATAGAAC	
lasI	Forward	CGATGGTTATGACGCACTCAGTCC	331
	Reverse	GATCATCATCTTCTCCACGCCTACG	
lasR	Forward	AGGACAGCCAGGACTACGAGAAC	321
	Reverse	GCAGGACCGACTCCATGAAACG	
rhlI	Forward	AATTGCTCTCTGAATCGCTGGAAGG	269
	Reverse	GGTTTCGCTGCACAGGTAGGC	
rhlR	Forward	AACAATTTGCTCAGCGTGCTTTCC	241
	Reverse	AGATGCTCAGGATGATGGCGATTTC	

Table S3 Primer sequences used for RT-qPCR

Genes	Direction	Sequences (5’-3’)
lasB	Forward	CGAAGCCATCACCGAAGTCAAGG
	Reverse	CAGCGATGTTGGCGACGAAATG
lasI	Forward	TGCGTGCTCAAGTGTTCAAGGAG
	Reverse	GGACTGAGTGCGTCATAACCATCG
lasR	Forward	GCTGGAACGCTCAAGTGGAAAATTG
	Reverse	TTCTCGTAGTCCTGGCTGTCCTTAG
rhlI	Forward	CTCTGAATCGCTGGAAGGGCTTTC
	Reverse	TTTGCGGATGGTCGAACTGGTC
rhlR	Forward	GAGCGATACCAGATGCAGAACTACG
	Reverse	TCCAGACCACCATTTCCGAGGAG
pqsA	Forward	GAAGTGAGCGAGGCGGTTCTG
	Reverse	CTGTTCGGCGAGATGCTGGTC
pqsR	Forward	TCGTTCTGCGATACGGTGAG
	Reverse	GCACTGGTTGAAGCGGGAG
16SrRNA	Forward	CTCTTCCTGGTCGGCGAAAAGC
	Reverse	GTTCGATACGCACCTGCCAGAG
